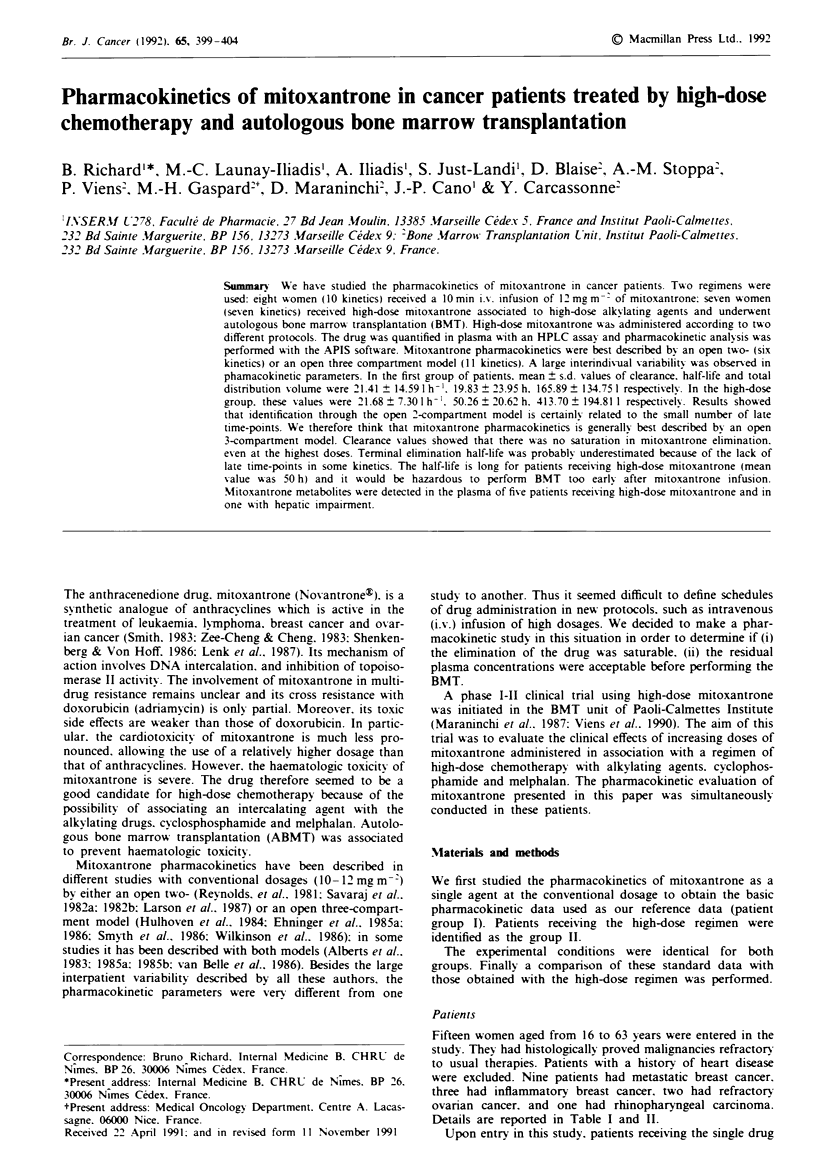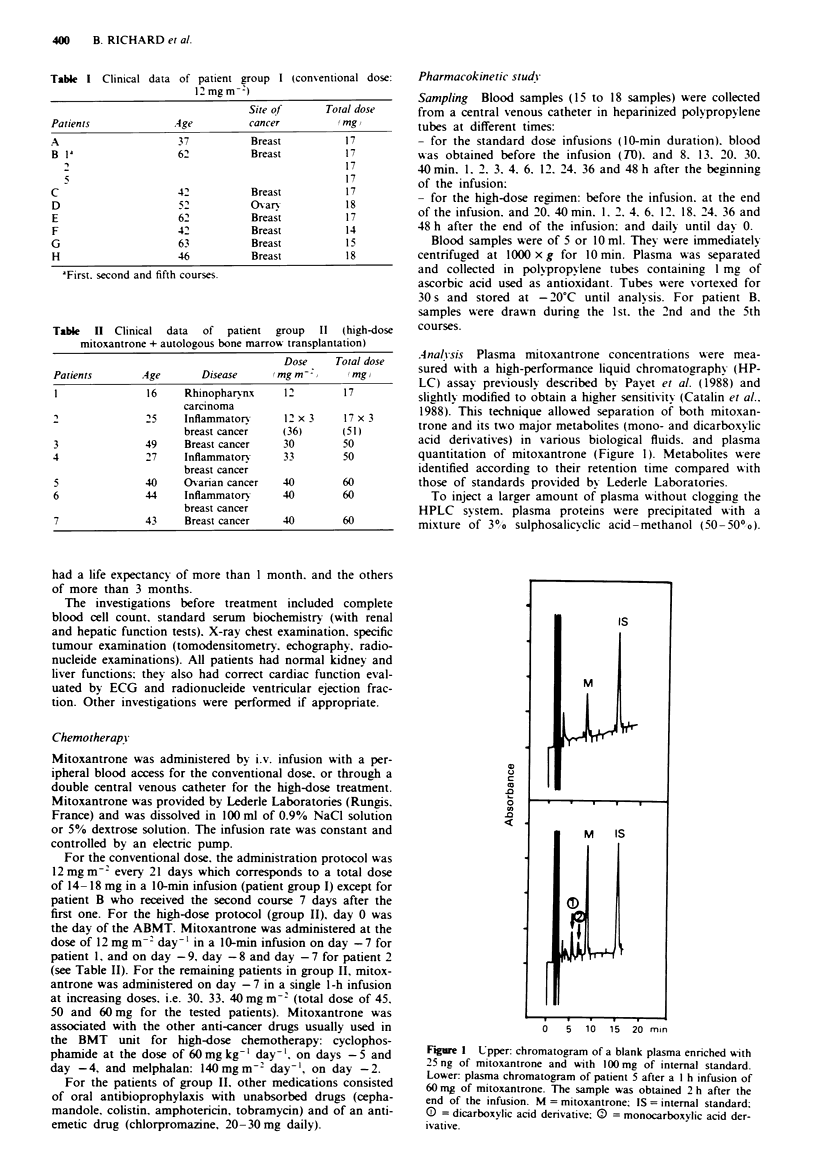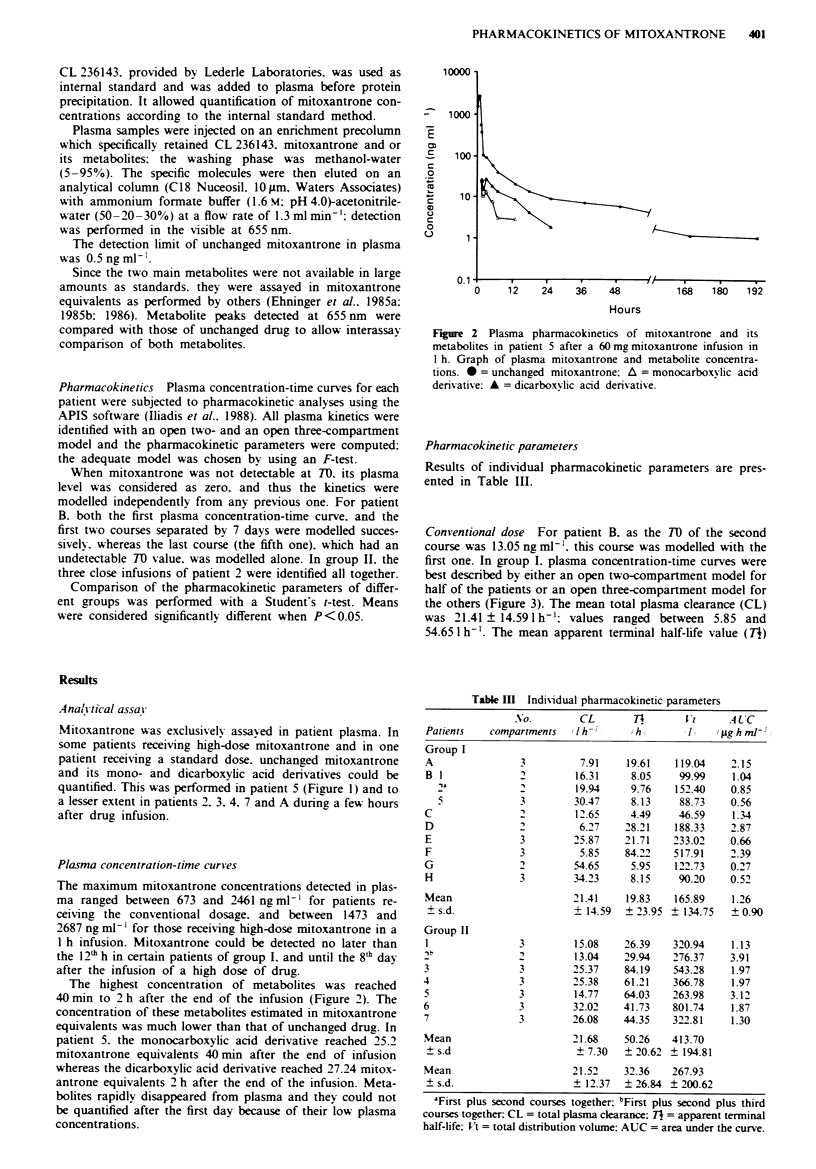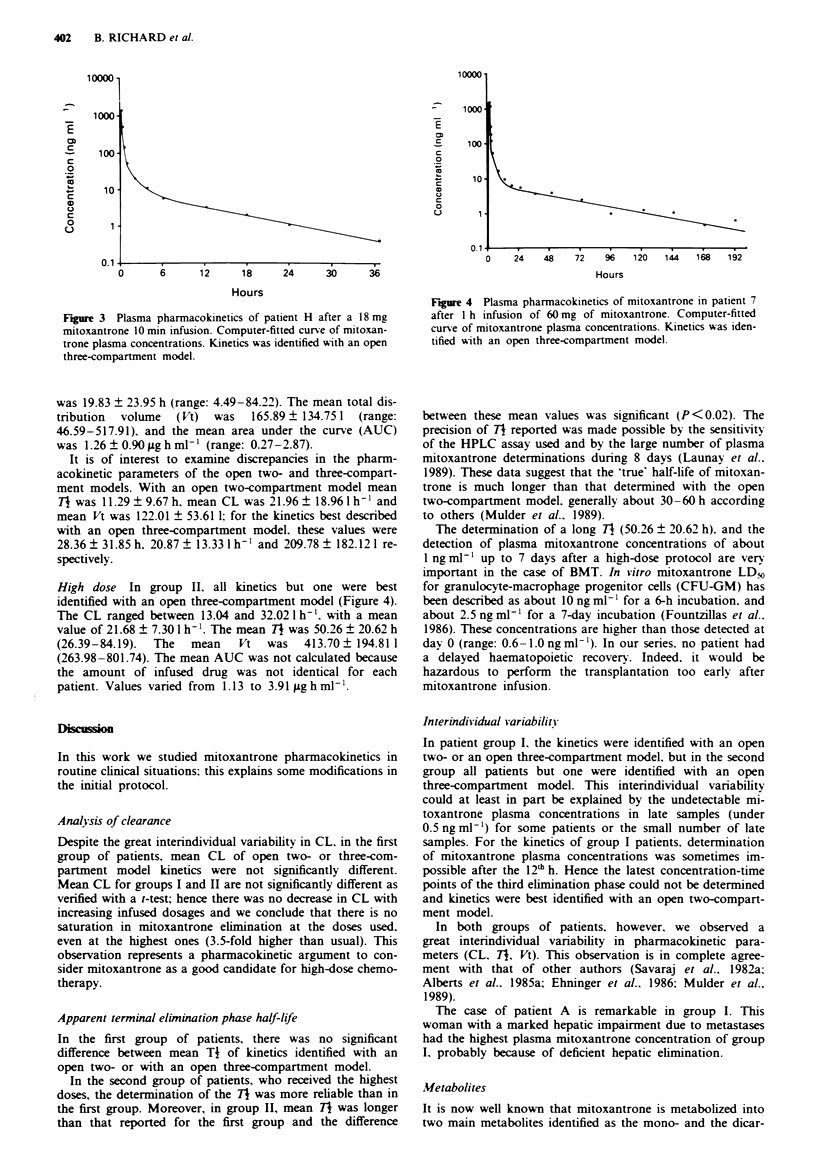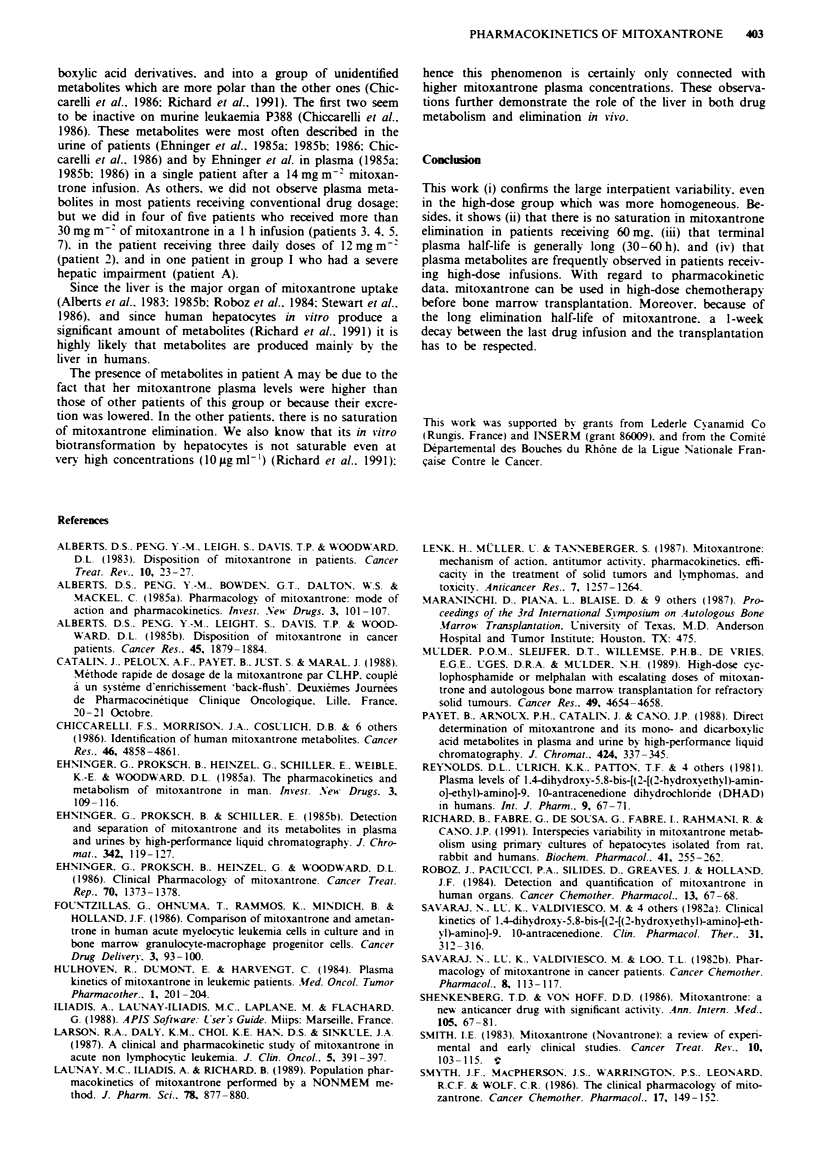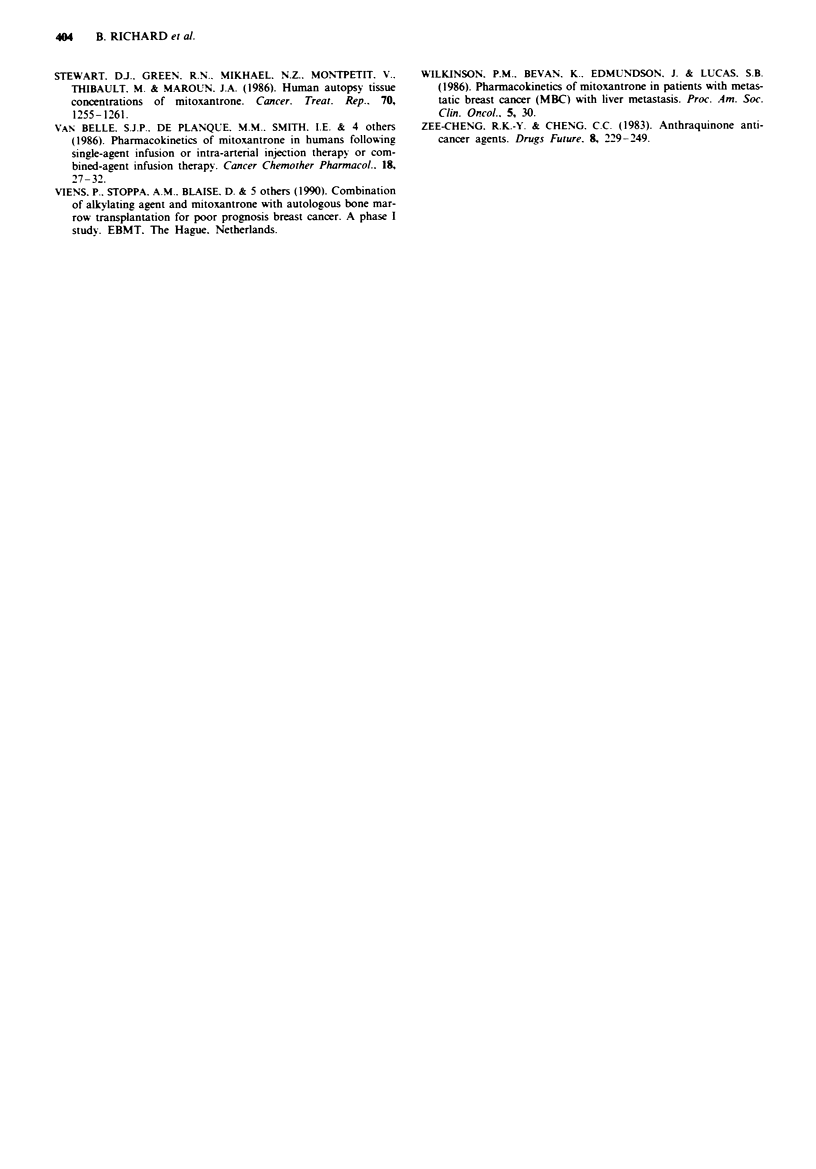# Pharmacokinetics of mitoxantrone in cancer patients treated by high-dose chemotherapy and autologous bone marrow transplantation.

**DOI:** 10.1038/bjc.1992.81

**Published:** 1992-03

**Authors:** B. Richard, M. C. Launay-Iliadis, A. Iliadis, S. Just-Landi, D. Blaise, A. M. Stoppa, P. Viens, M. H. Gaspard, D. Maraninchi, J. P. Cano

**Affiliations:** INSERM U278, Faculté de Pharmacie, Marseille, France.

## Abstract

We have studied the pharmacokinetics of mitoxantrone in cancer patients. Two regimens were used: eight women (10 kinetics) received a 10 min i.v. infusion of 12 mg m-2 of mitoxantrone; seven women (seven kinetics) received high-dose mitoxantrone associated to high-dose alkylating agents and underwent autologous bone marrow transplantation (BMT). High-dose mitoxantrone was administered according to two different protocols. The drug was quantified in plasma with an HPLC assay and pharmacokinetic analysis was performed with the APIS software. Mitoxantrone pharmacokinetics were best described by an open two- (six kinetics) or an open three compartment model (11 kinetics). A large interindivual variability was observed in pharmacokinetic parameters. In the first group of patients, mean +/- s.d. values of clearance, half-life and total distribution volume were 21.41 +/- 14.59 1 h-1, 19.83 +/- 23.95 h, 165.89 +/- 134.75 1 respectively. In the high-dose group, these values were 21.68 +/- 7.30 1 h-1, 50.26 +/- 20.62 h, 413.70 +/- 194.81 1 respectively. Results showed that identification through the open 2-compartment model is certainly related to the small number of late time-points. We therefore think that mitoxantrone pharmacokinetics is generally best described by an open 3-compartment model. Clearance values showed that there was no saturation in mitoxantrone elimination, even at the highest doses. Terminal elimination half-life was probably underestimated because of the lack of late time-points in some kinetics. The half-life is long for patients receiving high-dose mitoxantrone (mean value was 50 h) and it would be hazardous to perform BMT too early after mitoxantrone infusion. Mitoxantrone metabolites were detected in the plasma of five patients receiving high-dose mitoxantrone and in one with hepatic impairment.


Br. J. Cancer (1992), 65, 399-404 © Macmillan Press Ltd., 1992

### Pharmacokinetics of mitoxantrone in cancer patients treated by high-dose chemotherapy and autologous bone marrow transplantation

B. Richard[1]*, M.-C. Launay-Iliadis[1], A. Iliadis[1], S. Just-Landi[1], D. Blaise[2], A.-M. Stoppa[2], P. Viens[2], M.-H. Gaspard[2]†, D. Maraninchi[2], J.-P. Cano[1] & Y. Carcassonne[2]

[1]INSERM U278, Faculté de Pharmacie, 27 Bd Jean Moulin, 13385 Marseille Cédex 5, France and Institut Paoli-Calmettes, 232 Bd Sainte Marguerite, BP 156, 13273 Marseille Cédex 9; [2]Bone Marrow Transplantation Unit, Institut Paoli-Calmettes, 232 Bd Sainte Marguerite, BP 156, 13273 Marseille Cédex 9, France.

**Summary** We have studied the pharmacokinetics of mitoxantrone in cancer patients. Two regimens were used: eight women (10 kinetics) received a 10 min i.v. infusion of 12 mg m$^{-2}$ of mitoxantrone; seven women (seven kinetics) received high-dose mitoxantrone associated to high-dose alkylating agents and underwent autologous bone marrow transplantation (BMT). High-dose mitoxantrone was administered according to two different protocols. The drug was quantified in plasma with an HPLC assay and pharmacokinetic analysis was performed with the APIS software. Mitoxantrone pharmacokinetics were best described by an open two- (six kinetics) or an open three compartment model (11 kinetics). A large interindivual variability was observed in phamacokinetic parameters. In the first group of patients, mean ± s.d. values of clearance, half-life and total distribution volume were 21.41 ± 14.59 l h$^{-1}$, 19.83 ± 23.95 h, 165.89 ± 134.75 l respectively. In the high-dose group, these values were 21.68 ± 7.30 l h$^{-1}$, 50.26 ± 20.62 h, 413.70 ± 194.81 l respectively. Results showed that identification through the open 2-compartment model is certainly related to the small number of late time-points. We therefore think that mitoxantrone pharmacokinetics is generally best described by an open 3-compartment model. Clearance values showed that there was no saturation in mitoxantrone elimination, even at the highest doses. Terminal elimination half-life was probably underestimated because of the lack of late time-points in some kinetics. The half-life is long for patients receiving high-dose mitoxantrone (mean value was 50 h) and it would be hazardous to perform BMT too early after mitoxantrone infusion. Mitoxantrone metabolites were detected in the plasma of five patients receiving high-dose mitoxantrone and in one with hepatic impairment.

The anthracenedione drug, mitoxantrone (Novantrone®), is a synthetic analogue of anthracyclines which is active in the treatment of leukaemia, lymphoma, breast cancer and ovarian cancer (Smith, 1983; Zee-Cheng & Cheng, 1983; Shenkenberg & Von Hoff, 1986; Lenk *et al.*, 1987). Its mechanism of action involves DNA intercalation, and inhibition of topoisomerase II activity. The involvement of mitoxantrone in multidrug resistance remains unclear and its cross resistance with doxorubicin (adriamycin) is only partial. Moreover, its toxic side effects are weaker than those of doxorubicin. In particular, the cardiotoxicity of mitoxantrone is much less pronounced, allowing the use of a relatively higher dosage than that of anthracyclines. However, the haematologic toxicity of mitoxantrone is severe. The drug therefore seemed to be a good candidate for high-dose chemotherapy because of the possibility of associating an intercalating agent with the alkylating drugs, cyclosphosphamide and melphalan. Autologous bone marrow transplantation (ABMT) was associated to prevent haematologic toxicity.

Mitoxantrone pharmacokinetics have been described in different studies with conventional dosages (10-12 mg m$^{-2}$) by either an open two- (Reynolds, *et al.*, 1981; Savaraj *et al.*, 1982a; 1982b; Larson *et al.*, 1987) or an open three-compartment model (Hulhoven *et al.*, 1984; Ehninger *et al.*, 1985a; 1986; Smyth *et al.*, 1986; Wilkinson *et al.*, 1986): in some studies it has been described with both models (Alberts *et al.*, 1983; 1985a; 1985b; van Belle *et al.*, 1986). Besides the large interpatient variability described by all these authors, the pharmacokinetic parameters were very different from one study to another. Thus it seemed difficult to define schedules of drug administration in new protocols, such as intravenous (i.v.) infusion of high dosages. We decided to make a pharmacokinetic study in this situation in order to determine if (i) the elimination of the drug was saturable, (ii) the residual plasma concentrations were acceptable before performing the BMT.

A phase I-II clinical trial using high-dose mitoxantrone was initiated in the BMT unit of Paoli-Calmettes Institute (Maraninchi *et al.*, 1987; Viens *et al.*, 1990). The aim of this trial was to evaluate the clinical effects of increasing doses of mitoxantrone administered in association with a regimen of high-dose chemotherapy with alkylating agents, cyclophosphamide and melphalan. The pharmacokinetic evaluation of mitoxantrone presented in this paper was simultaneously conducted in these patients.

### Materials and methods

We first studied the pharmacokinetics of mitoxantrone as a single agent at the conventional dosage to obtain the basic pharmacokinetic data used as our reference data (patient group I). Patients receiving the high-dose regimen were identified as the group II.

The experimental conditions were identical for both groups. Finally a comparison of these standard data with those obtained with the high-dose regimen was performed.

### *Patients*

Fifteen women aged from 16 to 63 years were entered in the study. They had histologically proved malignancies refractory to usual therapies. Patients with a history of heart disease were excluded. Nine patients had metastatic breast cancer, three had inflammatory breast cancer, two had refractory ovarian cancer, and one had rhinopharyngeal carcinoma. Details are reported in Table I and II.

Upon entry in this study, patients receiving the single drug

Correspondence: Bruno Richard, Internal Medicine B, CHRU de Nîmes, BP 26, 30006 Nîmes Cédex, France.
*Present address: Internal Medicine B, CHRU de Nîmes, BP 26, 30006 Nîmes Cédex, France.
†Present address: Medical Oncology Department, Centre A. Lacassagne, 06000 Nice, France.
Received 22 April 1991; and in revised form 11 November 1991

Table I Clinical data of patient group I (conventional dose: 12 mg m$^{-2}$)

| Patients | Age | Site of cancer | Total dose (mg) |
|---|---|---|---|
| A | 37 | Breast | 17 |
| B 1[a] | 62 | Breast | 17 |
| 2 | | | 17 |
| 5 | | | 17 |
| C | 42 | Breast | 17 |
| D | 52 | Ovary | 18 |
| E | 62 | Breast | 17 |
| F | 42 | Breast | 14 |
| G | 63 | Breast | 15 |
| H | 46 | Breast | 18 |

[a]First, second and fifth courses.

Table II Clinical data of patient group II (high-dose mitoxantrone + autologous bone marrow transplantation)

| Patients | Age | Disease | Dose (mg m$^{-2}$) | Total dose (mg) |
|---|---|---|---|---|
| 1 | 16 | Rhinopharynx carcinoma | 12 | 17 |
| 2 | 25 | Inflammatory breast cancer | 12 × 3 (36) | 17 × 3 (51) |
| 3 | 49 | Breast cancer | 30 | 50 |
| 4 | 27 | Inflammatory breast cancer | 33 | 50 |
| 5 | 40 | Ovarian cancer | 40 | 60 |
| 6 | 44 | Inflammatory breast cancer | 40 | 60 |
| 7 | 43 | Breast cancer | 40 | 60 |

had a life expectancy of more than 1 month, and the others of more than 3 months.

The investigations before treatment included complete blood cell count, standard serum biochemistry (with renal and hepatic function tests), X-ray chest examination, specific tumour examination (tomodensitometry, echography, radionucleide examinations). All patients had normal kidney and liver functions; they also had correct cardiac function evaluated by ECG and radionucleide ventricular ejection fraction. Other investigations were performed if appropriate.

### Chemotherapy

Mitoxantrone was administered by i.v. infusion with a peripheral blood access for the conventional dose, or through a double central venous catheter for the high-dose treatment. Mitoxantrone was provided by Lederle Laboratories (Rungis, France) and was dissolved in 100 ml of 0.9% NaCl solution or 5% dextrose solution. The infusion rate was constant and controlled by an electric pump.

For the conventional dose, the administration protocol was 12 mg m$^{-2}$ every 21 days which corresponds to a total dose of 14–18 mg in a 10-min infusion (patient group I) except for patient B who received the second course 7 days after the first one. For the high-dose protocol (group II), day 0 was the day of the ABMT. Mitoxantrone was administered at the dose of 12 mg m$^{-2}$ day$^{-1}$ in a 10-min infusion on day −7 for patient 1, and on day −9, day −8 and day −7 for patient 2 (see Table II). For the remaining patients in group II, mitoxantrone was administered on day −7 in a single 1-h infusion at increasing doses, i.e. 30, 33, 40 mg m$^{-2}$ (total dose of 45, 50 and 60 mg for the tested patients). Mitoxantrone was associated with the other anti-cancer drugs usually used in the BMT unit for high-dose chemotherapy: cyclophosphamide at the dose of 60 mg kg$^{-1}$ day$^{-1}$, on days −5 and day −4, and melphalan: 140 mg m$^{-2}$ day$^{-1}$, on day −2.

For the patients of group II, other medications consisted of oral antibioprophylaxis with unabsorbed drugs (cephamandole, colistin, amphotericin, tobramycin) and of an anti-emetic drug (chlorpromazine, 20–30 mg daily).

### Pharmacokinetic study

*Sampling* Blood samples (15 to 18 samples) were collected from a central venous catheter in heparinized polypropylene tubes at different times:

- for the standard dose infusions (10-min duration), blood was obtained before the infusion (T0), and 8, 13, 20, 30, 40 min, 1, 2, 3, 4, 6, 12, 24, 36 and 48 h after the beginning of the infusion;
- for the high-dose regimen: before the infusion, at the end of the infusion, and 20, 40 min, 1, 2, 4, 6, 12, 18, 24, 36 and 48 h after the end of the infusion; and daily until day 0.

Blood samples were of 5 or 10 ml. They were immediately centrifuged at 1000 × *g* for 10 min. Plasma was separated and collected in polypropylene tubes containing 1 mg of ascorbic acid used as antioxidant. Tubes were vortexed for 30 s and stored at −20°C until analysis. For patient B, samples were drawn during the 1st, the 2nd and the 5th courses.

*Analysis* Plasma mitoxantrone concentrations were measured with a high-performance liquid chromatography (HPLC) assay previously described by Payet et al. (1988) and slightly modified to obtain a higher sensitivity (Catalin et al., 1988). This technique allowed separation of both mitoxantrone and its two major metabolites (mono- and dicarboxylic acid derivatives) in various biological fluids, and plasma quantitation of mitoxantrone (Figure 1). Metabolites were identified according to their retention time compared with those of standards provided by Lederle Laboratories.

To inject a larger amount of plasma without clogging the HPLC system, plasma proteins were precipitated with a mixture of 3% sulphosalicyclic acid–methanol (50–50%).

Figure 1 Upper: chromatogram of a blank plasma enriched with 25 ng of mitoxantrone and with 100 mg of internal standard. Lower: plasma chromatogram of patient 5 after a 1 h infusion of 60 mg of mitoxantrone. The sample was obtained 2 h after the end of the infusion. M = mitoxantrone; IS = internal standard; ① = dicarboxylic acid derivative; ② = monocarboxylic acid derivative.

CL 236143, provided by Lederle Laboratories, was used as internal standard and was added to plasma before protein precipitation. It allowed quantification of mitoxantrone concentrations according to the internal standard method.

Plasma samples were injected on an enrichment precolumn which specifically retained CL 236143, mitoxantrone and or its metabolites; the washing phase was methanol-water (5–95%). The specific molecules were then eluted on an analytical column (C18 Nuceosil, 10 µm, Waters Associates) with ammonium formate buffer (1.6 M; pH 4.0)-acetonitrile-water (50–20–30%) at a flow rate of 1.3 ml min$^{-1}$; detection was performed in the visible at 655 nm.

The detection limit of unchanged mitoxantrone in plasma was 0.5 ng ml$^{-1}$.

Since the two main metabolites were not available in large amounts as standards, they were assayed in mitoxantrone equivalents as performed by others (Ehninger *et al.*, 1985a; 1985b; 1986). Metabolite peaks detected at 655 nm were compared with those of unchanged drug to allow interassay comparison of both metabolites.

*Pharmacokinetics* Plasma concentration-time curves for each patient were subjected to pharmacokinetic analyses using the APIS software (Iliadis *et al.*, 1988). All plasma kinetics were identified with an open two- and an open three-compartment model and the pharmacokinetic parameters were computed; the adequate model was chosen by using an *F*-test.

When mitoxantrone was not detectable at *T*0, its plasma level was considered as zero, and thus the kinetics were modelled independently from any previous one. For patient B, both the first plasma concentration-time curve, and the first two courses separated by 7 days were modelled successively, whereas the last course (the fifth one), which had an undetectable *T*0 value, was modelled alone. In group II, the three close infusions of patient 2 were identified all together.

Comparison of the pharmacokinetic parameters of different groups was performed with a Student's *t*-test. Means were considered significantly different when $P < 0.05$.

### Results

### *Analytical assay*

Mitoxantrone was exclusively assayed in patient plasma. In some patients receiving high-dose mitoxantrone and in one patient receiving a standard dose, unchanged mitoxantrone and its mono- and dicarboxylic acid derivatives could be quantified. This was performed in patient 5 (Figure 1) and to a lesser extent in patients 2, 3, 4, 7 and A during a few hours after drug infusion.

### *Plasma concentration-time curves*

The maximum mitoxantrone concentrations detected in plasma ranged between 673 and 2461 ng ml$^{-1}$ for patients receiving the conventional dosage, and between 1473 and 2687 ng ml$^{-1}$ for those receiving high-dose mitoxantrone in a 1 h infusion. Mitoxantrone could be detected no later than the 12$^{th}$ h in certain patients of group I, and until the 8$^{th}$ day after the infusion of a high dose of drug.

The highest concentration of metabolites was reached 40 min to 2 h after the end of the infusion (Figure 2). The concentration of these metabolites estimated in mitoxantrone equivalents was much lower than that of unchanged drug. In patient 5, the monocarboxylic acid derivative reached 25.2 mitoxantrone equivalents 40 min after the end of infusion whereas the dicarboxylic acid derivative reached 27.24 mitoxantrone equivalents 2 h after the end of the infusion. Metabolites rapidly disappeared from plasma and they could not be quantified after the first day because of their low plasma concentrations.

**Figure 2** Plasma pharmacokinetics of mitoxantrone and its metabolites in patient 5 after a 60 mg mitoxantrone infusion in 1 h. Graph of plasma mitoxantrone and metabolite concentrations. ● = unchanged mitoxantrone; Δ = monocarboxylic acid derivative; ▲ = dicarboxylic acid derivative.

### *Pharmacokinetic parameters*

Results of individual pharmacokinetic parameters are presented in Table III.

*Conventional dose* For patient B, as the *T*0 of the second course was 13.05 ng ml$^{-1}$, this course was modelled with the first one. In group I, plasma concentration-time curves were best described by either an open two-compartment model for half of the patients or an open three-compartment model for the others (Figure 3). The mean total plasma clearance (CL) was 21.41 ± 14.59 l h$^{-1}$; values ranged between 5.85 and 54.65 l h$^{-1}$. The mean apparent terminal half-life value ($T\frac{1}{2}$)

**Table III** Individual pharmacokinetic parameters

| *Patients* | *No. compartments* | *CL (l h$^{-1}$)* | *T½ (h)* | *Vt (l)* | *AUC (µg h ml$^{-1}$)* |
|---|---|---|---|---|---|
| Group I | | | | | |
| A | 3 | 7.91 | 19.61 | 119.04 | 2.15 |
| B 1 | 2 | 16.31 | 8.05 | 99.99 | 1.04 |
| 2[a] | 2 | 19.94 | 9.76 | 152.40 | 0.85 |
| 5 | 3 | 30.47 | 8.13 | 88.73 | 0.56 |
| C | 2 | 12.65 | 4.49 | 46.59 | 1.34 |
| D | 2 | 6.27 | 28.21 | 188.33 | 2.87 |
| E | 3 | 25.87 | 21.71 | 233.02 | 0.66 |
| F | 3 | 5.85 | 84.22 | 517.91 | 2.39 |
| G | 2 | 54.65 | 5.95 | 122.73 | 0.27 |
| H | 3 | 34.23 | 8.15 | 90.20 | 0.52 |
| Mean ± s.d. | | 21.41 ± 14.59 | 19.83 ± 23.95 | 165.89 ± 134.75 | 1.26 ± 0.90 |
| Group II | | | | | |
| 1 | 3 | 15.08 | 26.39 | 320.94 | 1.13 |
| 2[b] | 2 | 13.04 | 29.94 | 276.37 | 3.91 |
| 3 | 3 | 25.37 | 84.19 | 543.28 | 1.97 |
| 4 | 3 | 25.38 | 61.21 | 366.78 | 1.97 |
| 5 | 3 | 14.77 | 64.03 | 263.98 | 3.12 |
| 6 | 3 | 32.02 | 41.73 | 801.74 | 1.87 |
| 7 | 3 | 26.08 | 44.35 | 322.81 | 1.30 |
| Mean ± s.d | | 21.68 ± 7.30 | 50.26 ± 20.62 | 413.70 ± 194.81 | |
| Mean ± s.d. | | 21.52 ± 12.37 | 32.36 ± 26.84 | 267.93 ± 200.62 | |

[a]First plus second courses together; [b]First plus second plus third courses together; CL = total plasma clearance; $T\frac{1}{2}$ = apparent terminal half-life; *V*t = total distribution volume; AUC = area under the curve.

**Figure 3** Plasma pharmacokinetics of patient H after a 18 mg mitoxantrone 10 min infusion. Computer-fitted curve of mitoxantrone plasma concentrations. Kinetics was identified with an open three-compartment model.

**Figure 4** Plasma pharmacokinetics of mitoxantrone in patient 7 after 1 h infusion of 60 mg of mitoxantrone. Computer-fitted curve of mitoxantrone plasma concentrations. Kinetics was identified with an open three-compartment model.

was 19.83 ± 23.95 h (range: 4.49–84.22). The mean total distribution volume (*V*t) was 165.89 ± 134.75 l (range: 46.59–517.91), and the mean area under the curve (AUC) was 1.26 ± 0.90 μg h $ml^{-1}$ (range: 0.27–2.87).

It is of interest to examine discrepancies in the pharmacokinetic parameters of the open two- and three-compartment models. With an open two-compartment model mean *T*½ was 11.29 ± 9.67 h, mean CL was 21.96 ± 18.96 l $h^{-1}$ and mean *V*t was 122.01 ± 53.61 l; for the kinetics best described with an open three-compartment model, these values were 28.36 ± 31.85 h, 20.87 ± 13.33 l $h^{-1}$ and 209.78 ± 182.12 l respectively.

*High dose* In group II, all kinetics but one were best identified with an open three-compartment model (Figure 4). The CL ranged between 13.04 and 32.02 l $h^{-1}$, with a mean value of 21.68 ± 7.30 l $h^{-1}$. The mean *T*½ was 50.26 ± 20.62 h (26.39–84.19). The mean *V*t was 413.70 ± 194.81 l (263.98–801.74). The mean AUC was not calculated because the amount of infused drug was not identical for each patient. Values varied from 1.13 to 3.91 μg h $ml^{-1}$.

### Discussion

In this work we studied mitoxantrone pharmacokinetics in routine clinical situations; this explains some modifications in the initial protocol.

### *Analysis of clearance*

Despite the great interindividual variability in CL, in the first group of patients, mean CL of open two- or three-compartment model kinetics were not significantly different. Mean CL for groups I and II are not significantly different as verified with a *t*-test; hence there was no decrease in CL with increasing infused dosages and we conclude that there is no saturation in mitoxantrone elimination at the doses used, even at the highest ones (3.5-fold higher than usual). This observation represents a pharmacokinetic argument to consider mitoxantrone as a good candidate for high-dose chemotherapy.

### *Apparent terminal elimination phase half-life*

In the first group of patients, there was no significant difference between mean T½ of kinetics identified with an open two- or with an open three-compartment model.

In the second group of patients, who received the highest doses, the determination of the *T*½ was more reliable than in the first group. Moreover, in group II, mean *T*½ was longer than that reported for the first group and the difference between these mean values was significant ($P<0.02$). The precision of *T*½ reported was made possible by the sensitivity of the HPLC assay used and by the large number of plasma mitoxantrone determinations during 8 days (Launay *et al.*, 1989). These data suggest that the 'true' half-life of mitoxantrone is much longer than that determined with the open two-compartment model, generally about 30–60 h according to others (Mulder *et al.*, 1989).

The determination of a long *T*½ (50.26 ± 20.62 h), and the detection of plasma mitoxantrone concentrations of about 1 ng $ml^{-1}$ up to 7 days after a high-dose protocol are very important in the case of BMT. *In vitro* mitoxantrone $LD_{50}$ for granulocyte-macrophage progenitor cells (CFU-GM) has been described as about 10 ng $ml^{-1}$ for a 6-h incubation, and about 2.5 ng $ml^{-1}$ for a 7-day incubation (Fountzillas *et al.*, 1986). These concentrations are higher than those detected at day 0 (range: 0.6–1.0 ng $ml^{-1}$). In our series, no patient had a delayed haematopoietic recovery. Indeed, it would be hazardous to perform the transplantation too early after mitoxantrone infusion.

### *Interindividual variability*

In patient group I, the kinetics were identified with an open two- or an open three-compartment model, but in the second group all patients but one were identified with an open three-compartment model. This interindividual variability could at least in part be explained by the undetectable mitoxantrone plasma concentrations in late samples (under 0.5 ng $ml^{-1}$) for some patients or the small number of late samples. For the kinetics of group I patients, determination of mitoxantrone plasma concentrations was sometimes impossible after the $12^{th}$ h. Hence the latest concentration-time points of the third elimination phase could not be determined and kinetics were best identified with an open two-compartment model.

In both groups of patients, however, we observed a great interindividual variability in pharmacokinetic parameters (CL, *T*½, *V*t). This observation is in complete agreement with that of other authors (Savaraj *et al.*, 1982a; Alberts *et al.*, 1985a; Ehninger *et al.*, 1986; Mulder *et al.*, 1989).

The case of patient A is remarkable in group I. This woman with a marked hepatic impairment due to metastases had the highest plasma mitoxantrone concentration of group I, probably because of deficient hepatic elimination.

### *Metabolites*

It is now well known that mitoxantrone is metabolized into two main metabolites identified as the mono- and the dicarboxylic acid derivatives, and into a group of unidentified metabolites which are more polar than the other ones (Chiccarelli *et al.*, 1986; Richard *et al.*, 1991). The first two seem to be inactive on murine leukaemia P388 (Chiccarelli *et al.*, 1986). These metabolites were most often described in the urine of patients (Ehninger *et al.*, 1985a; 1985b; 1986; Chiccarelli *et al.*, 1986) and by Ehninger *et al.* in plasma (1985a; 1985b; 1986) in a single patient after a 14 mg m$^{-2}$ mitoxantrone infusion. As others, we did not observe plasma metabolites in most patients receiving conventional drug dosage; but we did in four of five patients who received more than 30 mg m$^{-2}$ of mitoxantrone in a 1 h infusion (patients 3, 4, 5, 7), in the patient receiving three daily doses of 12 mg m$^{-2}$ (patient 2), and in one patient in group I who had a severe hepatic impairment (patient A).

Since the liver is the major organ of mitoxantrone uptake (Alberts *et al.*, 1983; 1985b; Roboz *et al.*, 1984; Stewart *et al.*, 1986), and since human hepatocytes *in vitro* produce a significant amount of metabolites (Richard *et al.*, 1991) it is highly likely that metabolites are produced mainly by the liver in humans.

The presence of metabolites in patient A may be due to the fact that her mitoxantrone plasma levels were higher than those of other patients of this group or because their excretion was lowered. In the other patients, there is no saturation of mitoxantrone elimination. We also know that its *in vitro* biotransformation by hepatocytes is not saturable even at very high concentrations (10 µg ml$^{-1}$) (Richard *et al.*, 1991); hence this phenomenon is certainly only connected with higher mitoxantrone plasma concentrations. These observations further demonstrate the role of the liver in both drug metabolism and elimination *in vivo*.

### Conclusion

This work (i) confirms the large interpatient variability, even in the high-dose group which was more homogeneous. Besides, it shows (ii) that there is no saturation in mitoxantrone elimination in patients receiving 60 mg, (iii) that terminal plasma half-life is generally long (30–60 h), and (iv) that plasma metabolites are frequently observed in patients receiving high-dose infusions. With regard to pharmacokinetic data, mitoxantrone can be used in high-dose chemotherapy before bone marrow transplantation. Moreover, because of the long elimination half-life of mitoxantrone, a 1-week decay between the last drug infusion and the transplantation has to be respected.

This work was supported by grants from Lederle Cyanamid Co (Rungis, France) and INSERM (grant 86009), and from the Comité Départemental des Bouches du Rhône de la Ligue Nationale Française Contre le Cancer.

### References

ALBERTS, D.S., PENG, Y.-M., LEIGH, S., DAVIS, T.P. & WOODWARD, D.L. (1983). Disposition of mitoxantrone in patients. *Cancer Treat. Rev.*, **10**, 23–27.

ALBERTS, D.S., PENG, Y.-M., BOWDEN, G.T., DALTON, W.S. & MACKEL, C. (1985a). Pharmacology of mitoxantrone: mode of action and pharmacokinetics. *Invest. New Drugs*, **3**, 101–107.

ALBERTS, D.S., PENG, Y.-M., LEIGHT, S., DAVIS, T.P. & WOODWARD, D.L. (1985b). Disposition of mitoxantrone in cancer patients. *Cancer Res.*, **45**, 1879–1884.

CATALIN, J., PELOUX, A.F., PAYET, B., JUST, S. & MARAL, J. (1988). Méthode rapide de dosage de la mitoxantrone par CLHP, couplé à un système d'enrichissement 'back-flush'. Deuxièmes Journées de Pharmacocinétique Clinique Oncologique, Lille, France, 20–21 Octobre.

CHICCARELLI, F.S., MORRISON, J.A., COSULICH, D.B. & 6 others (1986). Identification of human mitoxantrone metabolites. *Cancer Res.*, **46**, 4858–4861.

EHNINGER, G., PROKSCH, B., HEINZEL, G., SCHILLER, E., WEIBLE, K.-E. & WOODWARD, D.L. (1985a). The pharmacokinetics and metabolism of mitoxantrone in man. *Invest. New Drugs*, **3**, 109–116.

EHNINGER, G., PROKSCH, B. & SCHILLER, E. (1985b). Detection and separation of mitoxantrone and its metabolites in plasma and urines by high-performance liquid chromatography. *J. Chromat.*, **342**, 119–127.

EHNINGER, G., PROKSCH, B., HEINZEL, G. & WOODWARD, D.L. (1986). Clinical Pharmacology of mitoxantrone. *Cancer Treat. Rep.*, **70**, 1373–1378.

FOUNTZILLAS, G., OHNUMA, T., RAMMOS, K., MINDICH, B. & HOLLAND, J.F. (1986). Comparison of mitoxantrone and ametantrone in human acute myelocytic leukemia cells in culture and in bone marrow granulocyte-macrophage progenitor cells. *Cancer Drug Delivery*, **3**, 93–100.

HULHOVEN, R., DUMONT, E. & HARVENGT, C. (1984). Plasma kinetics of mitoxantrone in leukemic patients. *Med. Oncol. Tumor Pharmacother.*, **1**, 201–204.

ILIADIS, A., LAUNAY-ILIADIS, M.C., LAPLANE, M. & FLACHARD, G. (1988). *APIS Software: User's Guide*. Miips: Marseille, France.

LARSON, R.A., DALY, K.M., CHOI, K.E. HAN, D.S. & SINKULE, J.A. (1987). A clinical and pharmacokinetic study of mitoxantrone in acute non lymphocytic leukemia. *J. Clin. Oncol.*, **5**, 391–397.

LAUNAY, M.C., ILIADIS, A. & RICHARD, B. (1989). Population pharmacokinetics of mitoxantrone performed by a NONMEM method. *J. Pharm. Sci.*, **78**, 877–880.

LENK, H., MÜLLER, U. & TANNEBERGER, S. (1987). Mitoxantrone: mechanism of action, antitumor activity, pharmacokinetics, efficacity in the treatment of solid tumors and lymphomas, and toxicity. *Anticancer Res.*, **7**, 1257–1264.

MARANINCHI, D., PIANA, L., BLAISE, D. & 9 others (1987). *Proceedings of the 3rd International Symposium on Autologous Bone Marrow Transplantation*. University of Texas, M.D. Anderson Hospital and Tumor Institute: Houston, TX: 475.

MULDER, P.O.M., SLEIJFER, D.T., WILLEMSE, P.H.B., DE VRIES, E.G.E., UGES, D.R.A. & MULDER, N.H. (1989). High-dose cyclophosphamide or melphalan with escalating doses of mitoxantrone and autologous bone marrow transplantation for refractory solid tumours. *Cancer Res.*, **49**, 4654–4658.

PAYET, B., ARNOUX, P.H., CATALIN, J. & CANO, J.P. (1988). Direct determination of mitoxantrone and its mono- and dicarboxylic acid metabolites in plasma and urine by high-performance liquid chromatography. *J. Chromat.*, **424**, 337–345.

REYNOLDS, D.L., ULRICH, K.K., PATTON, T.F. & 4 others (1981). Plasma levels of 1,4-dihydroxy-5,8-bis-[(2-[(2-hydroxyethyl)-amino]-ethyl)-amino]-9, 10-antracenedione dihydrochloride (DHAD) in humans. *Int. J. Pharm.*, **9**, 67–71.

RICHARD, B., FABRE, G., DE SOUSA, G., FABRE, I., RAHMANI, R. & CANO, J.P. (1991). Interspecies variability in mitoxantrone metabolism using primary cultures of hepatocytes isolated from rat, rabbit and humans. *Biochem. Pharmacol.*, **41**, 255–262.

ROBOZ, J., PACIUCCI, P.A., SILIDES, D., GREAVES, J. & HOLLAND, J.F. (1984). Detection and quantification of mitoxantrone in human organs. *Cancer Chemother. Pharmacol.*, **13**, 67–68.

SAVARAJ, N., LU, K., VALDIVIESCO, M. & 4 others (1982a). Clinical kinetics of 1,4-dihydroxy-5,8-bis-[(2-[(2-hydroxyethyl)-amino]-ethyl)-amino]-9, 10-antracenedione. *Clin. Pharmacol. Ther.*, **31**, 312–316.

SAVARAJ, N., LU, K., VALDIVIESCO, M. & LOO, T.L. (1982b). Pharmacology of mitoxantrone in cancer patients. *Cancer Chemother. Pharmacol.*, **8**, 113–117.

SHENKENBERG, T.D. & VON HOFF, D.D. (1986). Mitoxantrone: a new anticancer drug with significant activity. *Ann. Intern. Med.*, **105**, 67–81.

SMITH, I.E. (1983). Mitoxantrone (Novantrone): a review of experimental and early clinical studies. *Cancer Treat. Rev.*, **10**, 103–115.

SMYTH, J.F., MACPHERSON, J.S., WARRINGTON, P.S., LEONARD, R.C.F. & WOLF, C.R. (1986). The clinical pharmacology of mitozantrone. *Cancer Chemother. Pharmacol.*, **17**, 149–152.

STEWART, D.J., GREEN, R.N., MIKHAEL, N.Z., MONTPETIT, V., THIBAULT, M. & MAROUN, J.A. (1986). Human autopsy tissue concentrations of mitoxantrone. *Cancer. Treat. Rep.*, **70**, 1255–1261.

VAN BELLE, S.J.P., DE PLANQUE, M.M., SMITH, I.E. & 4 others (1986). Pharmacokinetics of mitoxantrone in humans following single-agent infusion or intra-arterial injection therapy or combined-agent infusion therapy. *Cancer Chemother Pharmacol.*, **18**, 27–32.

VIENS, P., STOPPA, A.M., BLAISE, D. & 5 others (1990). Combination of alkylating agent and mitoxantrone with autologous bone marrow transplantation for poor prognosis breast cancer. A phase I study. EBMT. The Hague, Netherlands.

WILKINSON, P.M., BEVAN, K., EDMUNDSON, J. & LUCAS, S.B. (1986). Pharmacokinetics of mitoxantrone in patients with metastatic breast cancer (MBC) with liver metastasis. *Proc. Am. Soc. Clin. Oncol.*, **5**, 30.

ZEE-CHENG, R.K.-Y. & CHENG, C.C. (1983). Anthraquinone anticancer agents. *Drugs Future*, **8**, 229–249.